# Molecular Mechanisms of Seizure Gating in Sleep-Related Epilepsy: Genes, Channels, Sleep Stages and Circadian Rhythm

**DOI:** 10.3390/cimb48090931

**Published:** 2026-09-11

**Authors:** Ozgun Yetkin, Betul Baykan, Marcin Zarowski

**Affiliations:** 1 Polysomnography and Sleep Research Unit, Department of Developmental Neurology, Poznan University of Medical Sciences, 60-355 Poznan, Poland; zarowski@ump.edu.pl; 2 Department of Neurology and Clinical Neurophysiology, EMAR Medical Center, 34367 Istanbul, Türkiye

**Keywords:** sleep-related hypermotor epilepsy, NREM sleep, channelopathies, thalamocortical circuits, seizure gating

## Abstract

Seizures in several epilepsy syndromes cluster within narrow windows of the sleep–wake cycle, even though the underlying molecular defects are present continuously. This narrative review asks how constitutive molecular pathologies give rise to state-dependent seizures. We integrated epilepsy genetics with sleep neurophysiology, drawing on literature retrieved from PubMed/MEDLINE across eleven concept blocks, and organized the evidence around four gene families: nicotinic acetylcholine receptor subunits, KCNT1, the GATOR1–mTOR complex, and thalamocortical calcium and HCN channels. Across these pathways, the genetic evidence ranges from well-established monogenic causation to susceptibility alleles of insufficient effect. We propose, as an integrative working model, that non-rapid eye movement (NREM) sleep converts continuous defects into state-bound seizures by strengthening thalamocortical coupling, withdrawing cholinergic tone and destabilizing arousal, with circadian phase adding a further layer of temporal control. In this model, sleep-state dependence may reflect the interaction between molecular lesions and vigilance states rather than the properties of any single gene. The strength of evidence for this link varies across gene families and is weakest for the GATOR1 pathway; much of the supporting evidence derives from animal, expression, and computational studies, whose extension to human syndromes remains inferential.

## 1. Introduction

Sleep and epilepsy have a close, bidirectional relationship grounded in state-dependent changes in cortical excitability. Seizures are not randomly distributed but cluster across the 24 h sleep–wake cycle. Gowers first classified epilepsies as diurnal, nocturnal or diffuse on clinical grounds [1]. Janz and Christian later introduced the concept of awakening epilepsies, establishing that the state of alertness from which a seizure arises carries diagnostic and pathophysiological information [2]. Contemporary neurophysiology confirms that the transition between vigilance states, rather than clock time, is the primary gating mechanism [3].

The International League Against Epilepsy (ILAE) classification published in 2022 incorporates sleep timing into syndrome definitions, most explicitly in sleep-related hypermotor epilepsy (SHE) and syndromes with spike–wave activation in sleep (SWAS) [4,5]. Four syndromes span the lifespan and severity spectrum: SHE [6]; self-limited epilepsy with centrotemporal spikes (SeLECTS) [7]; developmental and epileptic encephalopathy with spike–wave activation in sleep (DEE-SWAS), where sleep discharges drive functional regression [8]; and awakening-linked idiopathic generalized epilepsies prototyped by juvenile myoclonic epilepsy (JME) [9]. Transitions through non-rapid eye movement (NREM) sleep are the unifying permissive condition across these entities [3,10].

These syndromes are genetically informative in some reports. SHE was the first monogenic focal epilepsy prototype to be solved through pathogenic *CHRNA4* variants [11,12]. SHE has an estimated minimum prevalence of 1.8 per 100,000 and accounts for approximately 10% of drug-resistant focal epilepsies [6]. Prevalence and incidence estimates for all four syndromes are summarized in Table 1.

The landscape now includes further nicotinic subunits (*CHRNB2*, *CHRNA2*), *KCNT1*, GATOR1–mTORC1 regulators (*DEPDC5*, *NPRL2*, *NPRL3*), *GRIN2A*, *CNKSR2*, as well as thalamocortical channels [13,14,15,16,17]. Seizure timing is determined by the interaction between the underlying molecular lesion and the dynamic physiological state of NREM sleep [3,10].

Sleep-related epileptiform activity also interferes with NREM-dependent processes, including slow-wave homeostasis and spindle-dependent memory consolidation [8]. This is most striking in DEE-SWAS, where developmental regression emerges in temporal association with immense discharge burden [8,17]. The relationship may not be straightforwardly causal; in a small number of reported cases, reducing the spike–wave index was not accompanied by developmental recovery [18], although the evidence is limited.

Despite progress in both domains, the genetic and neurophysiological studies have largely emerged in parallel, and reviews have focused on gene catalogs rather than mechanistic integration [16]. This review integrates the available evidence in response to three questions.

1. Which molecular pathologies cause sleep-related epilepsy syndromes, and by what cellular mechanisms?
2. How does NREM sleep alter the circuits in which these proteins operate, such that seizures cluster within a narrow temporal window?
3. Does the relationship between molecular class and sleep-state dependence follow a systematic gradient, or is it gene-specific?

## 2. Materials and Methods

### 2.1. Review Design

The present review was designed as a narrative review integrating two parallel studies: the clinical and genetic characterization of sleep-related epilepsy syndromes, and the neurophysiology of NREM sleep, around mechanistic questions. The evidence base spans clinical cohorts and syndrome classifications, heterologous expression electrophysiology, rodent models and computational simulation and was, therefore, considered unsuitable for quantitative pooling. The manuscript was assessed by the authors against the six items of the Scale for the Assessment of Narrative Review Articles (SANRA) [19].

### 2.2. Information Sources and Search Strategy

**Figure 1:**
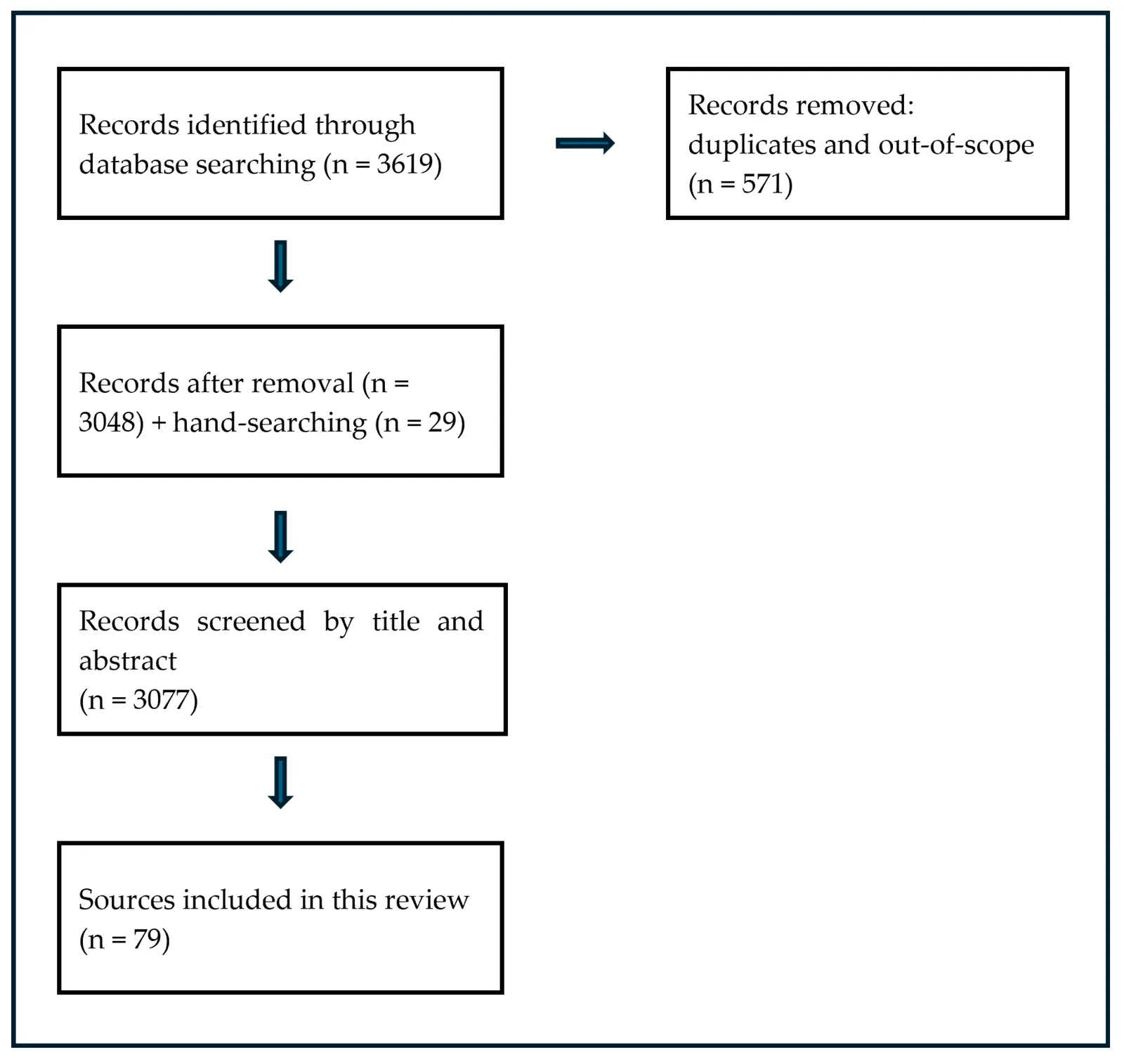
Flow diagram of study selection.

Records were retrieved from PubMed/MEDLINE and managed in EndNote X9 (Clarivate Analytics). The database searches identified 3619 records; after removal of duplicates and records outside the scope of the review (*n* = 571), 3048 unique records remained. Together with 29 records from hand-searching, 3077 records were screened by title and abstract (Figure 1). The search was organized into eleven concept blocks corresponding to the clinical syndromes, gene families and sleep-physiological processes addressed in this review: SHE; SeLECTS; SWAS; awakening-linked generalized epilepsies; nicotinic acetylcholine receptor genes; GATOR1–mTOR signaling; KCNT1; thalamocortical calcium and HCN channels; NREM sleep and the cyclic alternating pattern; sleep spindles and slow-wave activity; and circadian and clock-gene mechanisms. Within each block, syndrome names, gene symbols and physiological terms were combined with epilepsy and sleep terms using Boolean operators and MeSH headings were applied. Searches were conducted on 26–27 July 2026, with no date or language restrictions. Reference lists of retrieved articles were hand-searched, and ILAE position papers were retrieved directly. Because several of these syndromes have been renamed, superseded designations were searched alongside current terminology: nocturnal paroxysmal dystonia and autosomal dominant nocturnal frontal lobe epilepsy for SHE; benign epilepsy with centrotemporal spikes and Rolandic epilepsy for SeLECTS; and continuous spike–waves during slow sleep and electrical status epilepticus in sleep for SWAS. The search strings are provided in Supplementary Table S1.

### 2.3. Study Selection and Prioritization

The database searches yielded 3048 unique records after removal of duplicates and out-of-scope records, and a further 29 were identified through hand-searching of reference lists and ILAE position papers, for a total of 3077 records screened by title and abstract (Figure 1). Full texts of potentially relevant articles were retrieved and carefully analyzed.

Studies were included if they reported human genetic variants with functional or segregation evidence; characterized the syndrome definition, prevalence, penetrance or outcome in clinical cohorts or case series; examined state-dependent cortical excitability neurophysiologically; or provided mechanistic evidence from animal, in vitro, or computational work not obtainable in humans. Conference abstracts without full publication and publications not available in English were excluded.

Where sources overlapped or conflicted, evidence was prioritized in the following order: current ILAE position statements for nosology and syndrome definition; large unselected or systematically genotyped cohorts over selected pedigrees for prevalence and penetrance estimates; primary reports with functional validation over secondary descriptions of the same finding; human over animal or in vitro data; and, where a finding had been superseded, the most recent adequately powered study.

### 2.4. Data Synthesis

Findings were synthesized narratively in response to the three questions posed in the Introduction. Material is organized first by clinical syndrome, then by gene family and finally by physiological state, so that genetic and neurophysiological evidence can be compared within a common framework. No quantitative pooling was performed; effect sizes, proportions and statistical values are reported as given in the source.

## 3. Clinical Framing

Four syndrome groups illustrate the range of sleep-related seizure expression: SHE, SeLECTS, the encephalopathies with SWAS, and the awakening-linked generalized epilepsies. Their clinical, electrographic and genetic features are summarized in Table 1.

**Table 1:** Comparative clinical, electrographic and genetic features of sleep-related epileptic syndromes.

Syndrome	Onset Age	Prevalence/Incidence	Seizure Timing & Sleep Stage	Key Semiology & Pathological FeAtures	Principal Genes	Genetic Diagnostic Yield	Clinical Outcome & Comorbidity
SHE	Variable	Minimum 1.8/100,000; ~10% drug-resistant focal epilepsy	Predominantly N2; clustered, brief	Stereotyped hyperkinetic, tonic–dystonic; ~70% non-lesional	*DEPDC5*, *CHRNA4*, *KCNT1*, *NPRL2/3*	8.7% overall (19% familial, 7% sporadic) [13]	Cognitive/psychiatric comorbidities in subset; drug resistance in *DEPDC5* (~78%)
SeLECTS	3–13 years (peak 9–10)	~15% of childhood epilepsies; incidence 10–20/100,000	Near sleep onset or pre-waking NREM	Oropharyngolaryngeal, unilateral facial, speech arrest	*GRIN2A*, *ELP4*, *DEPDC5*, *KCNQ2/3*, 16p11.2	Low monogenic yield in typical cases; polygenic [20,21]	Self-limited seizures; group-level deficits in reading/language
DEE-SWAS/EE-SWAS	Childhood onset	Rare; precise prevalence not established	Continuous/activated throughout NREM	Regression temporally associated with SWAS; causal role unresolved	*GRIN2A* (34%), *ZEB2* (8%), *CNKSR2* (8%)	55% (DEE-SWAS) vs. 15% (EE-SWAS) [17]	Moderate-to-profound ID in 49% DEE-SWAS vs. 8% EE-SWAS
JME/Awakening	10–24 years	~9.3% of all epilepsies; 1–3/10,000	Within 2 h of waking, NREM activation	Morning myoclonic jerks, generalized tonic–clonic seizures on awakening	Polygenic; 15q13.3, 15q11.2, 16p13.11 CNVs	Microdeletions/CNVs in ~3% [9]	Lifelong seizure sensitivity; morning circadian vulnerability

Abbreviations: CNV, copy number variant; DEE-SWAS, developmental and epileptic encephalopathy with spike–wave activation in sleep; EE-SWAS, epileptic encephalopathy with spike–wave activation in sleep; ID, intellectual disability; JME, juvenile myoclonic epilepsy; N2, non-rapid eye movement sleep stage 2; NREM, non-rapid eye movement; SeLECTS, self-limited epilepsy with centrotemporal spikes; SHE, sleep-related hypermotor.

Estimates are drawn from heterogeneous study types and populations (population-based, surgical, and research cohorts) and are not directly comparable across syndromes; they are presented to characterize each syndrome individually rather than for direct quantitative comparison between syndromes.

### 3.1. Sleep-Related Hypermotor Epilepsy

SHE is the prototypical sleep-related focal epilepsy, defined by the ILAE as a disorder of variable onset age characterized by hyperkinetic or tonic–dystonic seizures arising predominantly from sleep, with genetic, structural or genetic–structural etiologies [5,6]. It was first described as ‘nocturnal paroxysmal dystonia’ [22], then as ‘autosomal dominant nocturnal frontal lobe epilepsy’ (ADNFLE) [23], and renamed to emphasize sleep gating rather than clock time while accommodating frequent extrafrontal onset [24,25]. The minimum prevalence is 1.8 per 100,000, accounting for roughly 10% of patients evaluated for surgery with a diagnosis of drug-resistant focal epilepsy [6].

Seizures are stereotyped, abrupt in onset and offset, brief, clustered, and predominantly arise during NREM stage 2 (N2) [25]. Because scalp electroencephalography (EEG) is often uninformative, diagnosis rests on clinical history and video documentation [3,25].

Around 70% of cases remain diagnosed as non-lesional [6]. Although historically conceptualized as a monogenic channelopathy, systematic genotyping of cohorts demonstrates that known pathogenic variants account for only 8.7% of patients overall (19% of familial and 7% of sporadic presentations) [13,26], with GATOR1 complex genes (5%) and *CHRNA4* (2.9%) dominant, and KCNT1 and *NPRL2* each at 1% [13].

**Figure 2:**
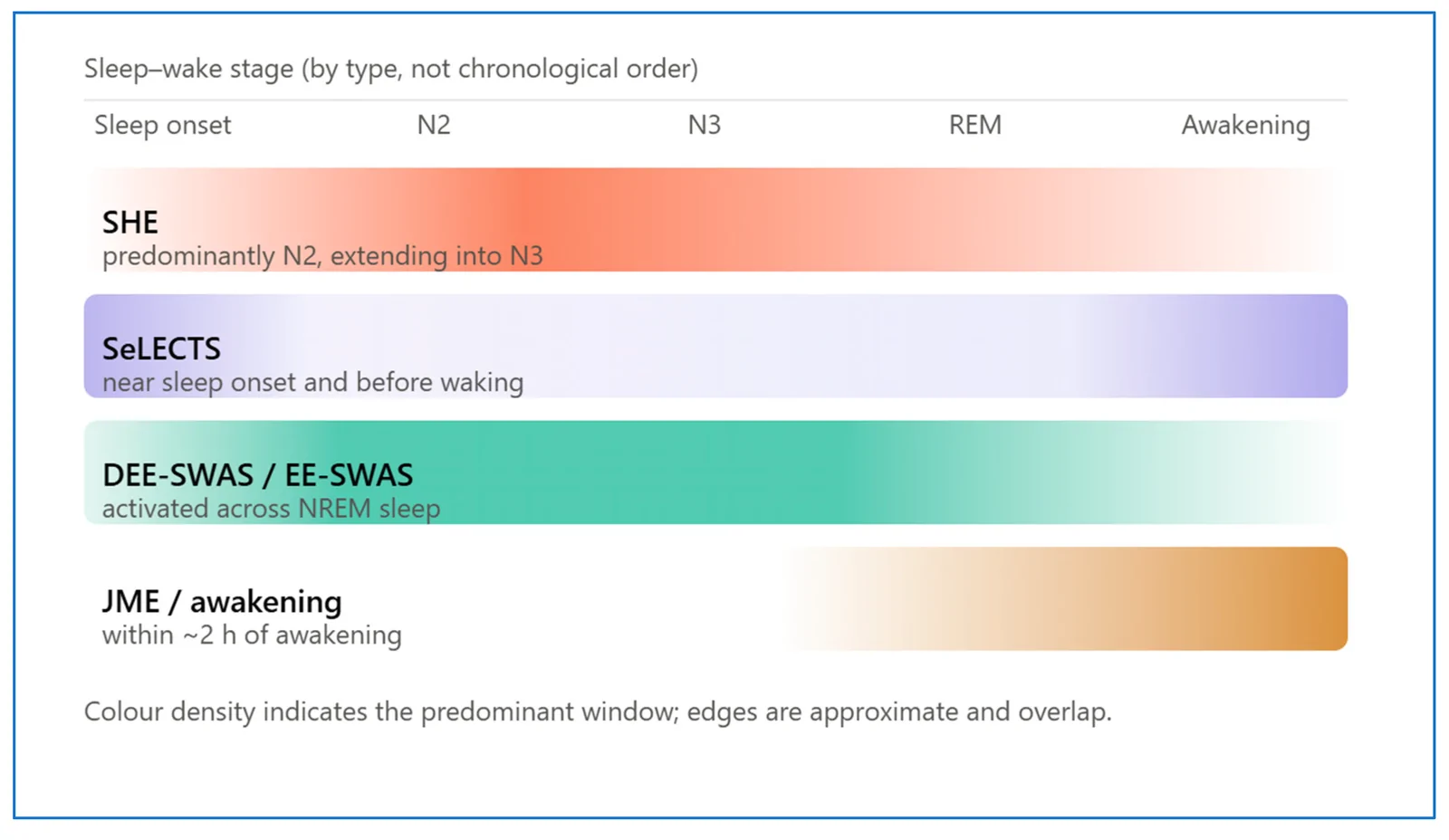
Sleep-stage distribution of seizure clustering across the four syndromes. Color density indicates the predominant window; stages are shown by type rather than chronological sequence within a sleep cycle, and edges are approximate and overlapping.

Figure 2 summarizes where each syndrome’s seizures cluster across the sleep–wake cycle, illustrating the state-dependent timing that this review examines at the molecular level in the following sections.

### 3.2. Self-Limited Epilepsy with Centrotemporal Spikes

SeLECTS, previously called benign epilepsy with centrotemporal spikes (BECTS) or Rolandic epilepsy, is the most common focal epilepsy syndrome of childhood, comprising roughly 15% of epilepsies in children aged 1–15 years, with an incidence of 10–20 per 100,000 [3,7]. Onset occurs between 3 and 13 years, peaking at 9–10 years, with about 75% beginning between 7 and 10 years [7,27]. The 2022 reclassification replaced “benign” with “self-limited” to signal that seizure remission does not exclude neurodevelopmental comorbidity [4,27].

Seizures arise from the lower Rolandic cortex representing the face and oropharynx, with unilateral facial sensorimotor and oropharyngolaryngeal symptoms, usually with preserved awareness; about half progress to focal-to-bilateral tonic–clonic seizures, predominantly in sleep [7]. Most arise from NREM sleep near sleep onset or before waking [3]. SeLECTS represents localized dysfunction of the perisylvian network, in which NREM slow oscillations provide a permissive substrate for focal spike synchronization [10].

Inheritance is non-Mendelian. The centrotemporal sharp-wave activity is transmitted as an autosomal-dominant trait with age-dependent penetrance, occurring in 2–4% of healthy children [28]. On the other hand, the clinical syndrome shows minimal monozygotic concordance and only 9.8% first-degree concordance, indicating polygenic susceptibility [20,21]. Heterozygous *GRIN2A* mutations are the commonest reported monogenic driver, rising to 17–20% in epilepsy–aphasia spectrum disorders [29,30,31]. Further known targets include *KCNQ2*, *KCNQ3*, *ELP4*, *GABRG2* and 16p11.2 microduplications [27,32]. Rare *DEPDC5* variants link a subset to GATOR1–mTORC1 hyperactivation, bridging SeLECTS and SHE [33]. SeLECTS is the mild end of the epilepsy–aphasia continuum, with 1.3–4.6% evolving into atypical forms, Landau–Kleffner syndrome or DEE-SWAS [7]. Meta-analysis shows group-level deficits in word reading (*d* = 0.71), language (*d* = 0.72–0.75) and phonological processing (*d* = 0.50) [34]. Since seizure frequency is low, seizures themselves are unlikely to account for these deficits, though the specific contribution of interictal discharges remains unresolved [35].

### 3.3. Developmental and Epileptic Encephalopathy with Spike–Wave Activation in Sleep (DEE-SWAS)

DEE-SWAS and its electroencephalographic marker, electrical status epilepticus during sleep (ESES), form the encephalopathic pole of the sleep-accentuated epilepsies [8]. The ILAE classification in 2022 separates DEE-SWAS, in which developmental impairment precedes SWAS, from EE-SWAS, in which development is normal until regression accompanies SWAS onset [4]. The pattern reflects secondary bilateral synchrony driven by a focal cortical generator [8].

Regression is not explained by clinical seizure burden alone. Eight of 101 individuals in one series had SWAS without ever having seizures [17], and in a systematic review of 172 published cases, 10 (6%) had no seizures, of whom eight carried *GRIN2A* variants [15]. This dissociation points away from overt seizures as the sole driver. Still, it does not exclude a contribution from sleep-potentiated interictal epileptiform activity, and both must be distinguished from the underlying developmental or genetic disorder itself.

In an unselected series of 91 patients, a cause was identified in 46%, genetic in 34% and structural in 13% [17]. Among patients admitted with SWAS and abnormal magnetic resonance imaging (MRI), thalamic injury was near-universal: 24 of 25 lesional patients, representing 37% of the whole cohort against 8% of controls, and lesions lateralized to the hemisphere generating the dominant pattern [36].

Diagnostic yield is higher at the developmental pole. In the core cohort of 91 patients, an etiology was identified in 66% of DEE-SWAS versus 28% of EE-SWAS cases, with a genetic cause in 55% versus 15%, respectively [17]. Causative genes fall into two brain co-expression modules, each positively correlated internally and negatively correlated with the other: an ion-channel module (*p* = 0.0002) and a transcriptional-regulation module (*p* = 0.04), with only *ATP1A2* and *NPRL2* outside both modules [17]. Across published cases, *GRIN2A* (34%), *ZEB2* (8%) and *CNKSR2* (8%) predominate, and genotype maps onto discharge topography: *GRIN2A* shows centrotemporal predilection, *CNKSR2* anterior bilateral discharges in 63%, and *ZEB2* frontocentral discharges in 92% [15]. Outcome diverges sharply: moderate-to-profound intellectual disability affected 49% of DEE-SWAS patients compared with 8% of EE-SWAS patients (*p* = 5.5 × 10^−6^) [17]. In two reported children, the spike–wave index fell substantially (from 84% to 16% and from 94% to 23%) without accompanying developmental change [18]; this observation is limited to two patients and should be regarded as anecdotal rather than as evidence that reducing the discharge cannot alter outcome.

Although *GRIN2A*, *ZEB2* and *CNKSR2* account for most genetically solved D/EE-SWAS cases, they are not examined here at the mechanistic depth given to the four gene families above. These genes act through glutamatergic signaling, transcriptional regulation and synaptic scaffolding, respectively, rather than through the channel- and mTORC1-level mechanisms that this review uses to link molecular lesion to sleep state; they are therefore discussed at the clinical and genetic level, while their mechanistic treatment is beyond the scope defined here.

ESES can also arise in other childhood epilepsy syndromes, including JME, where it does not invariably carry a poor prognosis [37]—a variability that, together with the overlap with idiopathic generalized epilepsy, links these sleep-accentuated encephalopathies to the awakening epilepsies considered next.

### 3.4. Awakening-Linked Generalized Epilepsies

The awakening epilepsies are prototyped by JME and epilepsy with generalized tonic–clonic seizures alone. Generalized tonic–clonic seizures typically occur within two hours of awakening. In contrast, myoclonic jerks occur frequently upon waking, facilitated by both sleep deprivation and forced early waking, with interictal epileptiform discharges activated during NREM sleep [3,9]. JME accounts for approximately 9.3% of epilepsies, with a prevalence of one to three per 10,000, onset typically between 10 and 24 years, and generalized tonic–clonic seizures in over 90% of patients [9].

Genetic architecture differs fundamentally from that of SHE or DEE-SWAS. Rare variants reported in *CACNB4*, *GABRA1*, *GABRD* and *EFHC1* have largely been discredited by larger cohorts [9]; polygenic susceptibility alleles and recurrent microdeletions instead drive risk at 15q13.3, 15q11.2 and 16p13.11. These copy number variants occur in about 3% of patients with idiopathic generalized epilepsy; the 15q13.3 microdeletion is found in 1% of patients against 0.02% of controls, raising risk substantially [9].

As NREM sleep progresses, brainstem cholinergic and monoaminergic withdrawal hyperpolarizes thalamocortical relay neurons, promoting burst firing and network hypersynchronization [3]. In drug-naive patients with JME, transcranial magnetic stimulation revealed reduced short and long-interval intracortical inhibition in the morning compared with the afternoon, an effect absent in focal epilepsy and controls; the authors attributed this to circadian variation in γ-aminobutyric acid (GABA)ergic inhibition driven by the suprachiasmatic nucleus, interacting with hyperexcitable thalamocortical networks [38].

## 4. Molecular and Cellular Mechanisms

**Figure 3:**
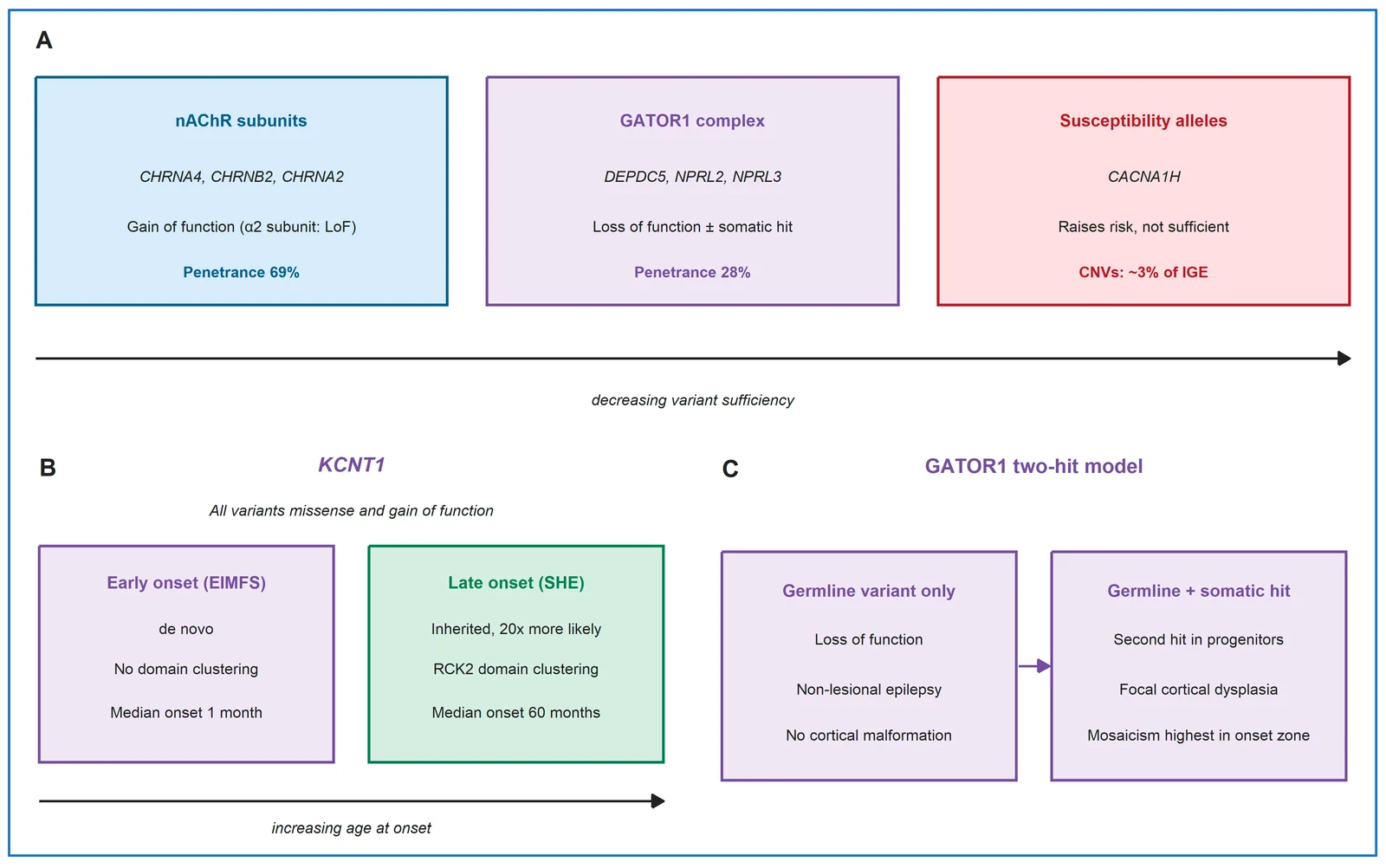
Genetic architecture across and within gene families in sleep-related epilepsies. (**A**) Cross-family gradient of variant sufficiency: nicotinic receptor variants are penetrant (69%), GATOR1 complex variants are less so (28%), and *CACNA1H* variants raise risk without being sufficient to cause epilepsy. (**B**) Within-gene recapitulation of the same gradient in *KCNT1*: early-onset cases are *de novo* with no domain clustering, late-onset cases are inherited with RCK2 clustering. (**C**) GATOR1 two-hit model: a germline loss-of-function variant alone causes non-lesional epilepsy, while an added somatic hit in brain progenitors causes focal cortical dysplasia.

The genes implicated in these syndromes fall into four families, whose molecular properties are summarized in Table 2 and whose genetic architecture is shown in Figure 3.

Abbreviations: *CACNA1H*, calcium voltage-gated channel subunit alpha1 H; *CHRNA2*/*CHRNA4*, cholinergic receptor nicotinic alpha 2/alpha 4 subunit; *CHRNB2*, cholinergic receptor nicotinic beta 2 subunit; CNV, copy number variant; *DEPDC5*, DEP domain-containing protein 5; EIMFS, epilepsy of infancy with migrating focal seizures; GATOR1, GAP activity toward Rags 1; IGE, idiopathic generalized epilepsy; *KCNT1*, potassium sodium-activated channel subfamily T member 1; LoF, loss of function; nAChR, neuronal nicotinic acetylcholine receptor; *NPRL2*/*NPRL3*, nitrogen permease regulator-like 2/3; RCK2, regulator of conductance for potassium domain 2; SHE, sleep-related hypermotor epilepsy.

### 4.1. Neuronal Nicotinic Acetylcholine Receptors (CHRNA4, CHRNB2, CHRNA2)

SHE was the first epilepsy syndrome for which a genetic cause was identified, through a missense mutation in *CHRNA4* [11]. *Variants in CHRNB2* [39] and *CHRNA2* [40] were subsequently identified. These discoveries established the concept of epilepsy as a channelopathy [40].

That concept has since narrowed. Only 10–12% of families carry a neuronal nicotinic acetylcholine receptor (nAChR) gene mutation [41], and in an unselected cohort, *CHRNA4* accounted for 2.9%, with no pathogenic *CHRNB2* or *CHRNA2* variants identified [13]. Penetrance is incomplete, at roughly 69% [23]. A single gene therefore explains neither most patients nor every carrier within a family [39,40].

The principal forebrain receptor is a pentamer of α4 and β2 subunits, assembled as (α4)_2_(β2)_3_ or (α4)_3_(β2)_2_. Five M2 segments line the pore and participate in gating [41]. Most disease-causing mutations fall in M2, making it a mutational hot spot; α4 S280F and S284L recur in unrelated families across several countries. β2 V287L also lies in M2, whereas α2 I279N sits in M1; this variant increases agonist sensitivity and was linked to familial epilepsy with nocturnal wandering and ictal fear [42]. By contrast, the *CHRNA2* variants associated with typical SHE cause loss of function even in heterozygotes [41].

Patients, however, are heterozygous, and in the simulated heterozygous state, mutations predominantly cause gain of function, usually by increasing sensitivity to acetylcholine, with variable effects on desensitization and little change in permeability [41]. β2 V287L illustrates this: the current decay constant rose from 65 ± 20 ms in wild-type to 8.2 ± 2 s in the mutant, while ion permeability was almost unchanged [39]. *CHRNA2* is the exception, since the variants found in typical cases cause loss of function even when heterozygous [41].

An overactive receptor can cause seizures by increasing, not reducing, inhibitory neurotransmission. Knock-in mice carrying α4 S280F showed recurrent seizures together with increased nicotine-evoked GABA release onto layer II/III pyramidal neurons [41]. The receptors responsible sit on inhibitory interneurons: in layer V of mouse frontal cortex, the α4 subunit was found on both parvalbumin-positive fast-spiking and somatostatin-positive cells, and nicotine increased the frequency of inhibitory postsynaptic currents through α4β2 receptors acting presynaptically [43]. Excessive synchronous inhibition appears to entrain pyramidal cells via rebound excitation, so seizures arise from excessive rather than insufficient GABAergic drive [43].

### 4.2. The GATOR1 Complex and mTORC1 Signaling Pathway (DEPDC5, NPRL2, NPRL3)

The mTOR pathway regulates cell growth, proliferation and migration in response to amino acids, insulin, growth factors and oxygen. Its amino acid-sensing branch is repressed by the GATOR1 complex, comprising DEPDC5, *NPRL2* and NPRL3 [44,45]. Loss-of-function variants in any subunit release this brake, thereby uncoupling mTOR activity from the cell’s metabolic state. The resulting mTORopathies produce irregular neuronal morphology, altered cortical lamination and increased excitability [44].

Exome sequencing found *DEPDC5* mutations in familial focal epilepsy with variable foci and in about 12% of families too small for formal diagnosis [46], with loss-of-function variants reported in 37% of families across a broader spectrum [47]. Screening 404 unrelated patients yielded five mutations in *NPRL2* and *NPRL3* along with 18 new *DEPDC5* mutations, establishing GATOR1 as the most significant known cause of familial focal epilepsy [48]. *NPRL3* was independently linked to familial cortical dysplasia [49] and to SHE [50].

Among 30 European families presenting as ADNFLE, *DEPDC5* mutations were found in 13% [51], whereas in an unselected SHE cohort, GATOR1 genes accounted for 5% [13]. Penetrance is low: only three of six carriers were affected in one *NPRL3* family [50], and 28% of carriers developed epilepsy in a large founder pedigree [52]. These observations may indicate that carrying a GATOR1 variant is not sufficient to cause epilepsy [53].

Part of the missing explanation is a second, somatic mutation. A germline variant alone lowers seizure threshold and produces non-lesional epilepsy, whereas a germline variant combined with a somatic second hit in brain progenitors produces focal cortical dysplasia. Analysis of postoperative human tissue confirmed this biallelic mechanism and revealed a mutation gradient, with mosaicism higher in the seizure-onset zone than in surrounding tissue [45]. In 80 patients with epilepsy surgery, germline, somatic and two-hit variants in *DEPDC5*, *TSC1*, and *TSC2* occurred exclusively in focal cortical dysplasia type 2 and hemimegalencephaly [54]. Modeling the somatic hit in mice reproduced the phenotype: impaired radial migration, doubled soma size, and phosphorylated ribosomal protein S6 (pS6) raised two- to fourfold, all of which were prevented by prenatal rapamycin [45].

Imaging findings are heterogeneous even within a single variant. In a founder pedigree carrying one *NPRL3* variant, imaging was available for 17 affected individuals: eight had normal brain structure, and nine had malformations of cortical development, eight of which were focal cortical dysplasias [52]. Clinically, GATOR1 variants carry a distinct signature: drug resistance reached 78% in *DEPDC5* patients, compared with roughly one-third in classical cohorts, and, reflecting the breadth of *DEPDC5*-related epilepsy beyond sleep-related presentations, diurnal seizures were reported in 60% [51,53].

Why these seizures remain confined to sleep is unresolved. Seizures in *NPRL3*-related disease arise immediately after arousal from N2 or N3 sleep [44], but no mechanism has been shown to link mTORC1 activity to the vigilance state. Unlike nicotinic receptors, where cholinergic tone provides a direct readout, GATOR1 variants act through a constitutive growth-signaling defect whose gating by sleep remains unexplained to date [44].

### 4.3. The Sodium-Activated Potassium Channel: KCNT1

*KCNT1* encodes a sodium-activated potassium channel subunit, also known as Slack, Slo2.2, or KCa4.1, and is the largest potassium channel subunit described to date [12]. It is expressed diffusely, mainly in the cerebellum, frontal cortex, brainstem and hippocampus [14]. Gating requires intracellular sodium concentrations near 40 mmol/L, so Slack operates within functional microdomains formed with sodium sources, including voltage-activated sodium channels and α-amino-3-hydroxy-5-methyl-4-isoxazolepropionic acid (AMPA) receptors [55].

The mutational picture is unusually uniform. Across 248 individuals, 64 distinct mutations were identified, all missense variants except for a single in-frame deletion, and every variant tested functionally showed gain of function. No clear pathogenic loss-of-function variant has been described, and truncating variants occur in healthy controls [14]. The magnitude can be extreme: p.Arg474His, the most severe known human variant, increases potassium currents up to 22-fold relative to wild-type [56].

Gain of function in a potassium channel might be expected to reduce excitability, and the epileptogenic mechanism is correspondingly indirect. Slack acts predominantly in inhibitory interneurons, where excess conductance suppresses the interneurons themselves and disinhibits downstream circuits; in excitatory cells, enhanced current accelerates repolarization and reduces sodium channel inactivation, permitting sustained high-frequency firing [16]. The relationship is not monotonic, however: Slack-knockout mice showed increased severity of kainic-acid-induced seizures, identifying the channel as a gatekeeper limiting seizure spread [55].

A study of 248 individuals with *KCNT1* variants found that most had early-onset DEEs, predominantly epilepsy of infancy with migrating focal seizures (EIMFS), and 53 had SHE, with a median onset of one month in early-onset cases against 60 months in late-onset sleep-related cases [14]. Late-onset syndromes were twenty times more likely to be inherited (95% CI 8–57), often through asymptomatic carrier parents. Sleep-related variants also cluster in the RCK2 domain (second regulator of conductance of potassium), whereas early-onset variants do not [14].

The severity of neurological involvement follows the same axis. In SHE, cognitive regression followed seizure onset in every patient and psychiatric comorbidities affected 57%, with normal MRI throughout [14]. At the early pole, nearly half of the children plateaued developmentally at onset, and 60% had abnormal MRI [57]. Mortality diverges sharply, with hazard ratios of 27 and 16 for the two early-onset syndromes relative to sleep-related epilepsy [14].

### 4.4. Thalamocortical Channels (CACNA1G, CACNA1H, CACNA1I, HCN1)

**Figure 4:**
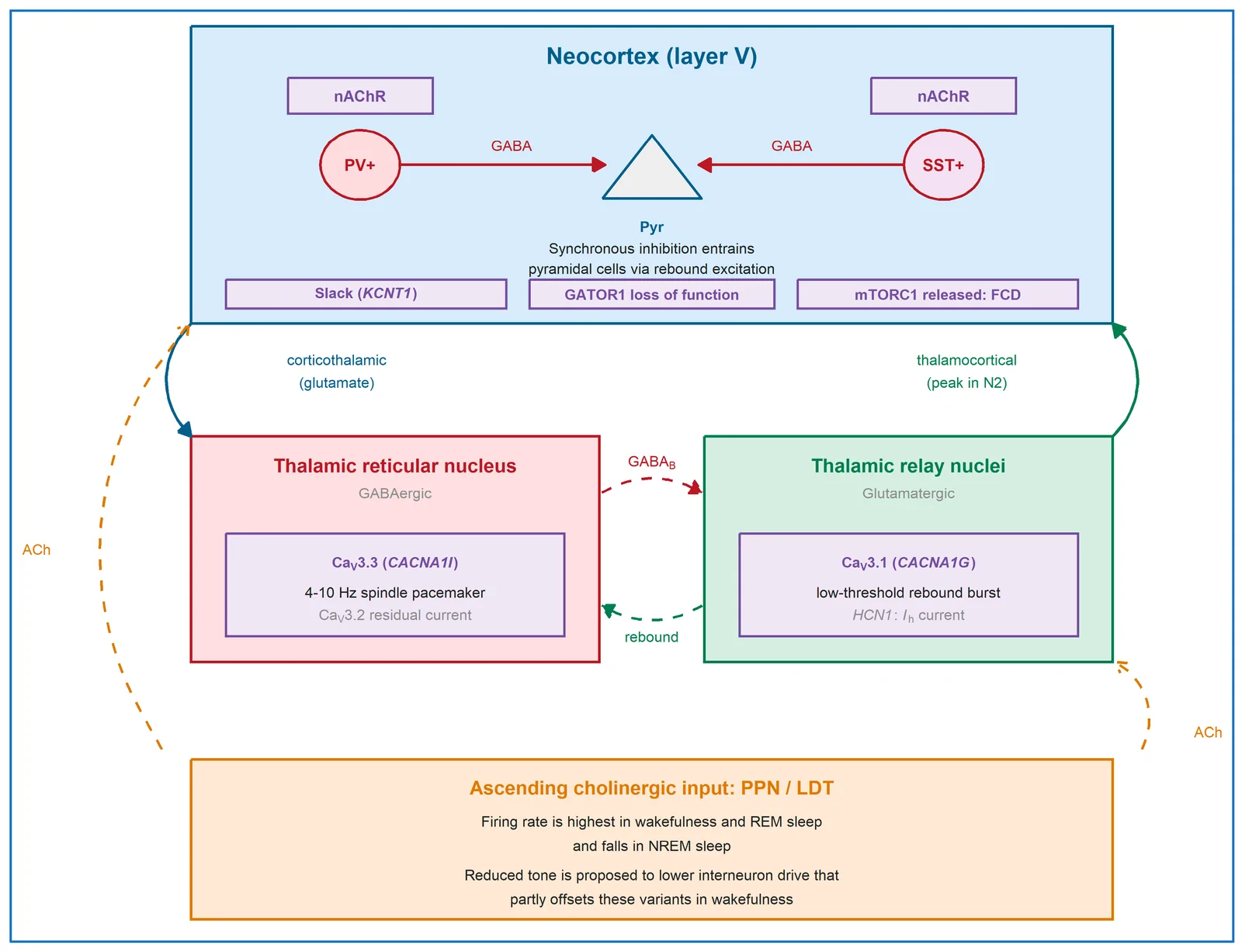
Thalamocortical circuit and molecular lesion overlay. Nicotinic receptor gain-of-function variants act on PV^+^ and SST^+^ interneurons in neocortical layer V, increasing GABA release onto pyramidal neurons and entraining them via rebound excitation. Slack (KCNT1) suppresses interneurons and accelerates pyramidal repolarization; GATOR1 loss-of-function releases mTORC1 in cortical progenitors, thereby generating focal cortical dysplasia. In the thalamic reticular nucleus, CaV3.3 (CACNA1I) paces 4–10 Hz oscillatory bursts. In thalamic relay nuclei, CaV3.1 (CACNA1G) generates the low-threshold rebound burst, while HCN1 carries the hyperpolarization-activated current Ih. The pedunculopontine (PPN) and laterodorsal tegmental (LDT) nuclei provide ascending cholinergic input, with firing rates highest in wakefulness and REM sleep and lower in NREM sleep; reduced cholinergic tone is proposed to lower the interneuron drive that partly offsets these variants during wakefulness.

The thalamocortical loop generates sleep spindles, the oscillations that characterize N2 [41]. The reticular nucleus provides the primary GABAergic input to the relay nuclei and bursts at spindle frequency, rhythmically hyperpolarizing relay neurons, which then fire rebound bursts that feed excitation back and sustain the oscillation [41]. The rebound burst depends on low-threshold calcium potentials activated once relay neurons are released from GABA-B-mediated hyperpolarization [58]. The four channel genes considered here carry different weights of evidence: *HCN1* and *CACNA1I* harbor *de novo* variants directly associated with epilepsy, *CACNA1H* is better regarded as a susceptibility allele than a sufficient cause, and *CACNA1G* has no established monogenic epilepsy variant and is included for its defined role in thalamocortical oscillation, characterized largely in animal models. The circuit and the cell-type localization of these proteins are shown in Figure 4.

Abbreviations: ACh, acetylcholine; CACNA1G/CACNA1H/CACNA1I, calcium voltage-gated channel subunits alpha1 G/H/I; CaV, voltage-gated calcium channel; GABA, γ-aminobutyric acid; GABAB, γ-aminobutyric acid type B receptor; HCN1, hyperpolarization-activated cyclic nucleotide-gated channel 1; Ih, hyperpolarization-activated cation current; KCNT1, potassium sodium-activated channel subfamily T member 1; LDT, laterodorsal tegmental nucleus; mTORC1, mechanistic target of rapamycin complex 1; N2, non-rapid eye movement sleep stage 2; nAChR, neuronal nicotinic acetylcholine receptor; NREM, non-rapid eye movement; PPN/PPT, pedunculopontine nucleus; PV^+^, parvalbumin-positive; Pyr, pyramidal neuron; REM, rapid eye movement; SST^+^, somatostatin-positive.

Four genes carry these currents: the T-type calcium channels *CACNA1G* (CaV3.1), *CACNA1H* (CaV3.2) and *CACNA1I* (CaV3.3), together with the hyperpolarization-activated channel *HCN1* [59]. Variants in thalamocortical channels alter the oscillatory machinery itself [59].

Channel expression is segregated by cell type. The reticular nucleus is rich in CaV3.3, while relay neurons rely on CaV3.1; when CaV3.3 is absent, the small residual current in reticular cells comes from CaV3.2 [59]. Deleting either channel disables a different component. Mice lacking CaV3.1 retained tonic firing in relay neurons but could not produce bursts, and their thalamus resisted spike–wave discharges evoked through GABA-B receptors [58]. Deleting CaV3.3 left reticular cells firing tonically but abolished their 4–10 Hz oscillatory bursts, degrading the network oscillation that produces spindles [59].

Variants in this family cause illness via two pathways. *CACNA1I* acts monogenically, with four heterozygous missense variants reported, producing phenotypes ranging from borderline cognitive impairment to severe developmental delay with epilepsy, each slowing activation, inactivation, and deactivation and shifting voltage dependence toward more negative potentials [60]. *HCN1* is comparable: *de novo* missense mutations in fever-sensitive drug-resistant epileptic encephalopathy showed marked but divergent effects on channel behavior [61], across a spectrum from neonatal DEE to benign generalized epilepsy [62].

On the other hand, *CACNA1H* behaves differently. Sequencing 118 children with absence epilepsy identified 12 heterozygous missense mutations in 14 patients, all at conserved residues and absent in 230 controls [63]; five of the former were functionally characterized [64]. A larger survey of 240 patients identified over 100 variants across childhood and juvenile absence, juvenile myoclonic and myoclonic-astatic epilepsies, febrile seizures and temporal lobe epilepsy, with nine of eleven tested variants altering channel behavior in a direction expected to increase calcium current [65]. Those authors concluded that such variants raise individual risk without being sufficient to cause epilepsy, and that because so many are individually rare, case–control association studies would be unlikely to detect *CACNA1H* at all [65].

Systematic genotyping converges on the same picture: ion channel subunits were the most common functional class among the 20 causative genes in D/EE-SWAS, and their brain co-expression exceeded that of 5000 randomly drawn gene sets of equal size [17]. The thalamocortical channels, therefore, mark the point at which single-gene causation gives way to co-expressed networks and susceptibility alleles, and they are the only genes considered here whose normal function constitutes the machinery of NREM sleep [66].

The strength of evidence differs markedly across these four pathways, both for disease causation and for the link to a specific vigilance state. Table 3 summarizes, for each pathway, the associated syndrome, the strength of human genetic evidence, the direct evidence for sleep-state dependence, the main experimental support, and the principal unresolved question.

## 5. NREM Sleep and the Gating of Seizures

The following synthesis is best read as a working model: the evidence for a role of NREM physiology is direct for some gene families and inferential for others, and the account below indicates which is which. Four properties of NREM sleep are relevant here: thalamocortical connectivity shifts across sleep stages, neuromodulatory tone declines, arousal oscillates, and small conductance changes are converted into synchronized discharge. Stereo-EEG recordings from 10 patients across seven brain regions, including the thalamus, show that connectivity varies by sleep stage. The thalamus was more connected to other regions during N2 and REM than during N3, whereas the cortex was more connected than the thalamus during N3 [69].

Neuromodulatory tone falls at the same time. The brainstem pedunculopontine and laterodorsal tegmental nuclei, which supply the thalamus, are active in wakefulness and REM sleep but almost silent in NREM [41]. For the nicotinic variants of Section 4.1, this inverts the usual logic of a channelopathy. High cholinergic tone in the awake brain drives local inhibition that partly offsets the mutation, thereby making wakefulness protective. Seizures then cluster in N2 during arousals, accompanied by bursts of ACh [41].

Within N2, arousal itself oscillates. In JME, the cyclic alternating pattern rate is elevated across N1, N2 and N3, in drug-naive patients and those on valproate alike, and discharges follow this oscillation, peaking in phase A and suppressed in phase B [70]. Among 107 patients with idiopathic generalized epilepsy, discharges overlapped K-complexes in 65.4% but sleep spindles in only 10.3%, and no clinical variable predicted this overlap [71]. The same principle separates SHE from disorders of arousal, in which phase A1 is reduced, and major motor events arise within N3 [72]. Modeling shows how the sleeping circuit converts single-channel defects into network pathology. Four separate manipulations each generated roughly 4 Hz discharges beginning in the neocortex: raised tonic GABA-A inhibition in first-order relay neurons, reduced cortical phasic inhibition, increased cortical AMPA function, and increased T-type conductance in higher-order relay neurons [66]. Reducing leak conductance instead gave 7 Hz discharges with spindles preserved, whereas increasing T-type conductance across reticular neurons produced only brief discharges insufficient for an ictal phenotype [66].

## 6. Circadian Modulation of Seizure Timing

Seizure susceptibility in epilepsy varies with time of day, and in some patients, seizures occur only during sleep [73,74]. Since sleep can begin at any clock time, this distribution implicates a second timing system. That system is the molecular clock, a transcription–translation feedback loop. BMAL1 and CLOCK form a heterodimer and activate transcription of the negative regulators, *PER* and *CRY* genes [75]. These gene families also regulate neuronal excitability and seizure susceptibility [73]. A systematic review screening 6364 records and including 26 studies found that most point in the same direction: expression of CLOCK and BMAL1 is reduced in several animal models, and knocking out these genes makes seizures more severe [74]. Reduced expression in an epileptic animal may be a consequence as much as a cause [76].

Rats kindled with pentylenetetrazole and then chronically sleep-deprived became more drug-resistant [77]. Hippocampal BMAL1 and PER2 fell, phosphorylated S6 rose, and P-glycoprotein increased. Experiments in endothelial cells and astrocytes showed that BMAL1 controls P-glycoprotein through the mTOR pathway, and the effect moved in either direction: overexpressing BMAL1 reduced drug resistance and lowered P-glycoprotein, while knocking it down produced drug resistance in normally sleeping rats [77].

P-glycoprotein is an efflux transporter at the blood–brain barrier. Circadian oscillations modulate the expression of such transporters, and higher expression is associated with a poorer response to antiseizure medication [75].

Glial mechanisms contribute independently: in a chemogenetic model, the same dose produced more severe seizures in the light phase than in the dark phase, and astrocyte-specific knockdown of the adrenergic α2 receptor worsened susceptibility only in the light phase, an effect partly reversed by an α2 agonist [78]. Circadian variation in threshold is therefore not purely neuronal; both GABA and glutamate show circadian modulation [73].

Modeling links this to the channels in Section 4. The introduction of a circadian component to the Epileptor-2 model adjusted both excitability and dosages to influence seizure onset and timing, thereby increasing efficacy during peak times of susceptibility [79].

These influences, NREM synchronization, arousal instability and circadian modulation, are best understood as overlapping and interacting processes rather than as a strict hierarchy; their relative contributions likely vary across syndromes and individuals.

## 7. Discussion

The initial three questions can now be examined, but with various levels of confidence.

Three things follow from this synthesis, and they should be distinguished. First, what the common framework genuinely integrates: across all four gene families, seizures are gated by vigilance state rather than occurring at random, and NREM sleep is the shared permissive condition—this state-dependence is the one property that unifies otherwise unrelated molecular lesions, and it is supported most directly for the nicotinic and *KCNT1*-related syndromes. Second, what remains gene-specific and is not explained by the shared framework: the cellular mechanism differs fundamentally between families—inhibitory hypersynchrony for nicotinic variants, channel gain-of-function for *KCNT1*, mTORC1 disinhibition and somatic dysplasia for GATOR1, and altered oscillation for thalamocortical channels—so the framework unifies the *timing* of seizures, not their *mechanism*. Third, where the evidence is contradictory or absent: the link between molecular lesion and sleep state is asserted but unproven for GATOR1, where nothing connects mTORC1 activity to vigilance state; the clock-gene literature is limited and partly conflicting; and whether spike–wave activation causes regression or merely marks it remains unresolved.

Which molecular pathologies cause sleep-related epilepsy syndromes, and by what cellular mechanisms? The mechanisms differ systematically by gene family. Nicotinic receptor variants act through interneurons, where mutant receptors increase GABA release onto pyramidal cells, so seizures arise from excessive synchronous inhibition rather than from too little [41,43]. *KCNT1* variants are almost uniformly missense and gain-of-function [14]. GATOR1 variants release the brake on mTORC1 and produce cortical dysplasia when a somatic second hit occurs in brain progenitors [45]. Thalamocortical channel variants alter the oscillation itself [59].

How does NREM sleep alter the circuits in which these proteins operate, such that seizures cluster within a narrow temporal window? Thalamic connectivity to cortex is greater in N2 and REM than in N3 [69]; cholinergic tone falls, removing the inhibition that protects the awake brain [41]; arousal oscillates, and discharges track that oscillation rather than sleep depth [71]; and the sleeping circuit converts single conductance changes into synchronized discharge, though which rhythm results depends on which conductance changes and where [66].

Does the relationship between molecular class and sleep-state dependence follow a systematic gradient, or is it gene-specific? Penetrance is high but incomplete for nicotinic receptor variants (Section 4.1), falling to 28% in a large GATOR1 pedigree [52], and disappears altogether for *CACNA1H*, whose variants raise risk without causing epilepsy and are so rare that association studies would miss the gene entirely [65]. The same gradient appears within *KCNT1* alone, where severe early-onset cases are de novo and milder late-onset sleep-related cases are inherited with incomplete penetrance [14].

Precision therapy has been harder than the genetics implied. Where the mechanism is understood, targeted treatment can be effective. Several mechanism-directed treatments have been explored, but they rest on very different levels of evidence and are best regarded as emerging directions rather than established options. In small case series, transdermal nicotine reduced seizures in CHRNA4 carriers, consistent with restoring the cholinergic tone disrupted by NREM withdrawal, and carbamazepine blocks the open nAChR channel at therapeutic concentrations [41]. Elsewhere, results have been mixed. Quinidine, now evaluated in three systematic reviews and a small controlled trial, has produced inconsistent results in KCNT1-related epilepsy, with the controlled trial finding no significant seizure reduction; the ketogenic diet outperformed it among patients with functional-domain variants [68]. Everolimus, in a small open-label series, reduced seizures in patients with DEPDC5 loss-of-function variants, although the single patient with an NPRL3 variant worsened [44,80]. Experimental antisense knockdown reduced seizures in two infants with KCNT1 p.Arg474, but caused hydrocephalus in both [56]. Network-level approaches are also under study: thalamocortical deep brain stimulation has produced meaningful seizure reduction in small series [81]. Across all of these, the evidence remains preliminary—small series, open-label observation, or experimental treatment—and none yet supports a clinical recommendation.

Three questions remain open. First, whether spike–wave activation is a cause or a marker. As noted in Section 3.3, the limited available evidence does not show that reducing the discharge restores development [18], but this rests on very few cases and remains unresolved. Second, state dependence in GATOR1 epilepsy has no molecular explanation. Seizures follow arousals from N2 or N3 [44], yet nothing links mTORC1 activity to vigilance state, though the BMAL1–mTOR axis offers a plausible starting point [77]. Third, the clock-gene literature remains limited and partly conflicting [74].

## 8. Limitations

Several limitations follow from the design. Records were retrieved from a single bibliographic database, so relevant work indexed elsewhere may have been missed. Although no language restriction was applied during retrieval, 166 non-English records were identified and could not be assessed, potentially introducing bias. Screening and selection were performed without duplicate independent review, and no protocol was pre-registered, in keeping with the narrative design. Several penetrance and prevalence estimates are based on founder- or referral-based cohorts and are likely to be revised as larger series are reported. Finally, much of the mechanistic evidence linking molecular lesions to vigilance state comes from animal, heterologous expression and computational studies, and its extension to human syndromes remains inferential.

## 9. Conclusions

Sleep-related epilepsies are not a collection of unrelated channelopathies that happen to occur at night. Across nicotinic receptors, *KCNT1*, the GATOR1 complex and the thalamocortical channels, the genetic architecture follows a gradient from constrained monogenic causation, through incomplete penetrance and somatic second hits, to susceptibility alleles insufficient on their own. We propose, as an integrative working model, that what these lesions share is not a pathway but a temporal window: each may become epileptogenic when NREM sleep reconfigures the circuit in which it sits, by strengthening thalamocortical coupling, withdrawing neuromodulatory tone and destabilizing arousal. The evidence for this state dependence is strongest for the nicotinic receptor and KCNT1-related syndromes and remains to be established for the GATOR1 pathway, where the link between mTORC1 signaling and vigilance state is not yet understood. On this model, state dependence may reflect the interaction between molecule and state rather than the properties of any individual gene—a framing that, if confirmed, would suggest directing diagnosis, prognosis and treatment at that interaction rather than at the genotype alone.

## Figures and Tables

**Table 2:** Molecular and cellular determinants across gene families implicated in sleep-related epileptic syndromes.

Gene/Complex	Protein/Unit	Functional Class	Variant Type & Direct Effect	Primary Cell Type of Action	Penetrance & Architecture	Associated Phenotypes
*CHRNA4/CHRNB2*	α4, β2 nAChR subunits	Ligand-gated cation channel	Missense (M2 hot spot); Gain-of-function (ACh hypersensitivity)	Cortical GABAergic interneurons (PV^+^, SST^+^)	Incomplete penetrance (69%)	SHE/ADNFLE
*CHRNA2*	α2 nAChR subunit	Ligand-gated cation channel	Heterozygous missense; loss-of-function in typical SHE (I279N is gain-of-function)	Cortical interneurons/circuits	Incomplete penetrance	SHE/ADNFLE
*DEPDC5/NPRL2/NPRL3*	GATOR1 complex subunits	mTORC1 amino acid-sensing repressor	Loss-of-function (germline ± somatic 2nd-hit)	Brain progenitors/pyramidal lineages	Low penetrance (~28%); 2nd-hit dictates FCD II	Familial focal epilepsy, SHE, FCD Type II
*KCNT1*	Slack/K_Na_1.1 subunit	Na^+^-activated K^+^ channel	Uniformly missense; Gain-of-function (up to 22-fold)	Interneurons (suppression) & Excitatory cells (fast repolarization)	Sporadic (*de novo*) in EIMFS; Incomplete in SHE	EIMFS (early onset), SHE (late onset)
*CACNA1I*	CaV3.3 subunit	Low-threshold T-type Ca^2+^ channel	Heterozygous missense; altered gating & voltage dependence	Thalamic reticular nucleus neurons	predominantly *de novo*; penetrance not established	Neurodevelopmental delay ± epilepsy
*CACNA1H*	CaV3.2 subunit	Low-threshold T-type Ca^2+^ channel	Missense variants; increased Ca^2+^ current	Thalamic reticular nucleus (residual current)	Incomplete/Susceptibility risk allele	Childhood absence, idiopathic generalized epilepsy, spectrum susceptibility
*CACNA1G*	CaV3.1 subunit	Low-threshold T-type Ca^2+^ channel	Knockout/loss-of-function (animal models)	Thalamic relay neurons	No established monogenic epilepsy variants; mechanistic evidence from mouse knockout	Absence seizure resistance (mouse knockout)
HCN1	HCN1 channel subunit	Hyperpolarization-activated cation channel	*De novo* missense; shifting activation/gating	Thalamocortical relay & cortical neurons	Predominantly *de novo*; penetrance not established	Early infantile epileptic encephalopathy to benign generalized

Abbreviations: ACh, acetylcholine; ADNFLE, autosomal dominant nocturnal frontal lobe epilepsy; Ca^2+^, calcium ion; CaV, voltage-gated calcium channel; EIMFS, epilepsy of infancy with migrating focal seizures; FCD II, focal cortical dysplasia type II; GABAergic, γ-aminobutyric acid-releasing; K^+^, potassium ion; mTORC1, mechanistic target of rapamycin complex 1; Na^+^, sodium ion; nAChR, neuronal nicotinic acetylcholine receptor; PV^+^, parvalbumin-positive; SHE, sleep-related hypermotor epilepsy; SST^+^, somatostatin-positive.

**Table 3:** Summary of the four molecular pathways in sleep-related epilepsy: associated syndrome, strength of human genetic evidence, direct evidence for sleep-state dependence, main experimental support, and the key unresolved question for each.

Gene/Pathway	Associated Syndrome	Strength of Human Genetic Evidence	Direct Evidence for Sleep-State Dependence	Main Experimental Support	Key Unresolved Question
Nicotinic acetylcholine receptors (*CHRNA4*, *CHRNB2*, *CHRNA2*)	SHE (ADNFLE)	Strong—established causal genes across multiple pedigrees [11,39]; incomplete penetrance (~69%) [23]	Strong clinically (seizures are almost exclusively sleep-bound); the link to cholinergic arousal is inferential	Altered ACh sensitivity in heterologous expression; rodent models; therapeutic response to nicotine in carriers [67]	Why do identical receptor defects generate seizures only in NREM, when cholinergic tone falls in several vigilance transitions?
*KCNT1* (Slack K^+^ channel)	SHE and EIMFS (within-gene spectrum)	Strong—recurrent gain-of-function variants [57]; genotype spans severe *de novo* to inherited sleep-related forms	Partial—sleep-bound seizures in the SHE end; no established mechanism tying channel gain-of-function to a specific vigilance state	Gain-of-function confirmed in expression systems; quinidine block in vitro; variable clinical response [68]	How one gain-of-function channel yields both an early encephalopathy and a sleep-confined focal epilepsy
GATOR1 complex (*DEPDC5*, *NPRL2*, *NPRL3*)	SHE and familial focal epilepsy; focal cortical dysplasia	Strong for disease causation; two-hit (germline + somatic) mechanism well supported [45]	Weak—the molecular link between mTORC1 dysregulation and vigilance state is unclear; sleep-state dependence is clinical, not mechanistically established	Loss-of-function releasing mTORC1 [48]; somatic second hits in resected dysplasia; mouse models	Whether and how mTORC1 signaling is coupled to NREM sleep, or whether the sleep pattern arises downstream
Thalamocortical Ca^2+^ and HCN channels (*CACNA1G*, *CACNA1H*, *HCN1*)	Awakening-linked and generalized epilepsies: roles in normal oscillations	Mixed—*CACNA1H* is a susceptibility factor rather than a sufficient cause [65]; *HCN1* is *de novo* but rare [61,62]; oscillatory role better established than disease causation [66]	Indirect—channels constitute the machinery of NREM spindles and slow waves, so a state link is expected but is largely inferred from physiology	Computational and rodent models of thalamocortical oscillation; limited human genetic penetrance data	Whether these channels drive seizures or mainly set the oscillatory context in which other lesions become epileptogenic

Abbreviations: ACh, acetylcholine; ADNFLE, autosomal dominant nocturnal frontal lobe epilepsy; EIMFS, epilepsy of infancy with migrating focal seizures; HCN, hyperpolarization-activated cyclic nucleotide-gated; mTORC1, mechanistic target of rapamycin complex 1; NREM, non-rapid eye movement; SHE, sleep-related hypermotor epilepsy.

## Data Availability

No new datasets were generated or analyzed in this study. Data sharing is not applicable to this article.

## Supplementary Materials

The following supporting information can be downloaded at: https://www.mdpi.com/article/10.3390/cimb48090931/s1.

## Abbreviations

The following abbreviations are used in this manuscript:

ACh	Acetylcholine
ADNFLE	Autosomal Dominant Nocturnal Frontal Lobe Epilepsy
AMPA	α-Amino-3-hydroxy-5-methyl-4-isoxazolepropionic Acid
BECTS	Benign Epilepsy with Centrotemporal Spikes
BMAL1	Basic Helix-Loop-Helix ARNT Like 1
Ca_V_	Voltage-Gated Calcium Channel
CLOCK	Clock Circadian Regulator
CNV	Copy Number Variant
CRY	Cryptochrome
DEE	Developmental and Epileptic Encephalopathy
DEE-SWAS	Developmental and epileptic encephalopathy with spike–wave activation in sleep
EE-SWAS	Epileptic Encephalopathy with Spike–Wave Activation in Sleep
EEG	Electroencephalography
EIMFS	Epilepsy of Infancy with Migrating Focal Seizures
ESES	Electrical Status Epilepticus During Sleep
FCD II	Focal Cortical Dysplasia Type II
GABA	γ-Aminobutyric Acid
GATOR1	GAP Activity Toward Rags 1 Complex
HCN1	Hyperpolarization-Activated Cyclic Nucleotide-Gated Channel 1
*I*_h_	Hyperpolarization-Activated Cation Current
ILAE	International League Against Epilepsy
JME	Juvenile Myoclonic Epilepsy
MRI	Magnetic Resonance Imaging
mTORC1	Mechanistic Target of Rapamycin Complex 1
nAChR	Neuronal Nicotinic Acetylcholine Receptor
NREM	Non-Rapid Eye Movement Sleep
nRT	Thalamic Reticular Nucleus
pS6	Phosphorylated Ribosomal Protein S6
PV	Parvalbumin
Pyr	Pyramidal Neuron
RCK2	Regulator of Conductance for Potassium Domain 2
REM	Rapid Eye Movement Sleep
SeLECTS	Self-Limited Epilepsy with Centrotemporal Spikes
SHE	Sleep-related hypermotor epilepsy
SST	Somatostatin
SWAS	Spike–Wave Activation in Sleep
