# Supplementary material for: Molecular Mechanisms of Seizure Gating in Sleep-Related Epilepsy: Genes, Channels, Sleep Stages and Circadian Rhythm
Source: Curr Issues Mol Biol. 2026 Sep 11;48(9):931. doi: 10.3390/cimb48090931 (PMC13605621; doi:10.3390/cimb48090931)
Supplement: Supplementary file 1 [file cimb-48-00931-s001.zip › cimb-4530822-supplementary.pdf]

Supplementary Table S1. PubMed/MEDLINE search strings for the eleven concept blocks, with the number of records retrieved into the reference library. Database searches were conducted on 26–27 July 2026, with no date or language restrictions. Duplicate and out-of-scope records were subsequently removed (see Figure 1).

Concept block	PubMed/MEDLINE search string	n
Sleep-related hypermotor epilepsy (SHE)	("sleep-related hypermotor epilepsy"[Title] OR "nocturnal frontal lobe epilepsy"[Title] OR "autosomal dominant nocturnal frontal lobe epilepsy"[Title] OR "ADNFLE"[Title] OR "nocturnal paroxysmal dystonia"[Title]) AND ("gene"[Title/Abstract] OR "genetic"[Title/Abstract] OR "mutation"[Title/Abstract] OR "variant"[Title/Abstract] OR "channel"[Title/Abstract] OR "receptor"[Title/Abstract] OR "molecular"[Title/Abstract])	127
SeLECTS / centrotemporal spikes	("self-limited epilepsy with centrotemporal spikes"[Title] OR "benign epilepsy with centrotemporal spikes"[Title] OR "benign childhood epilepsy with centrotemporal spikes"[Title] OR "benign rolandic epilepsy"[Title] OR "rolandic epilepsy"[Title] OR "centrotemporal spike*"[Title]) AND ("gene*"[Title/Abstract] OR "mutation"[Title/Abstract] OR "variant*"[Title/Abstract] OR "GRIN2A"[Title/Abstract] OR "channel"[Title/Abstract])	191
CSWS / ESES (spike-wave activation in sleep)	("continuous spike-wave*"[Title/Abstract] OR "continuous spikes and waves"[Title/Abstract] OR "electrical status epilepticus"[Title/Abstract] OR "ESES"[Title/Abstract] OR "CSWS"[Title/Abstract] OR "spike-wave activation in sleep"[Title/Abstract] OR "Landau-Kleffner"[Title/Abstract] OR "DEE-SWAS"[Title/Abstract] OR "EE-SWAS"[Title/Abstract]) AND ("mutation"[Title/Abstract] OR "variant*"[Title/Abstract] OR "gene"[Title/Abstract] OR "GRIN2A"[Title/Abstract] OR "CNKSR2"[Title/Abstract])	249
Awakening-linked generalized epilepsies	("epilepsy with generalized tonic-clonic seizures on awakening"[Title] OR "awakening epilepsy"[Title] OR "seizures on awakening"[Title] OR ("juvenile myoclonic epilepsy"[Title] AND ("circadian"[Title/Abstract] OR "sleep"[Title/Abstract] OR "awakening"[Title/Abstract] OR "chronotype"[Title/Abstract] OR "diurnal"[Title/Abstract])))	89
Nicotinic acetylcholine receptor genes	("CHRNA4"[Title/Abstract] OR "CHRNB2"[Title/Abstract] OR "CHRNA2"[Title/Abstract] OR "nicotinic acetylcholine receptor"[Title/Abstract]) AND ("epilep*"[Title/Abstract] OR "seizure*"[Title/Abstract])	347
KCNT1	("KCNT1"[Title/Abstract] OR "Slack channel"[Title/Abstract] OR "KNa1.1"[Title/Abstract]) AND ("epilep*"[Title/Abstract] OR "seizure*"[Title/Abstract])	200
GATOR1-mTOR signaling	("DEPDC5"[Title/Abstract] OR "NPRL2"[Title/Abstract] OR "NPRL3"[Title/Abstract]) AND ("epilep*"[Title/Abstract] OR "seizure*"[Title/Abstract])	227
Thalamocortical calcium and HCN channels	("CACNA1G"[Title/Abstract] OR "CACNA1H"[Title/Abstract] OR "CACNA1I"[Title/Abstract] OR "HCN1"[Title/Abstract]) AND ("epilep*"[Title/Abstract] OR "seizure*"[Title/Abstract])	227
NREM sleep and cyclic alternating pattern	(("NREM sleep"[Title/Abstract] OR "non-REM sleep"[Title/Abstract] OR "non-rapid eye movement sleep"[Title/Abstract]) AND ("epilep*"[Title] OR "seizure*"[Title])) OR ("cyclic alternating pattern"[Title/Abstract] AND ("epilep*"[Title/Abstract] OR "seizure*"[Title/Abstract]))	397
Sleep spindles and slow-wave activity	("sleep spindle*"[Title/Abstract] OR "slow wave activity"[Title/Abstract] OR "K-complex*"[Title/Abstract] OR "thalamocortical oscillation*"[Title/Abstract] OR "spindle oscillation*"[Title/Abstract] OR "slow-wave sleep"[Title]) AND ("epilep*"[Title/Abstract] OR "seizure*"[Title/Abstract])	732
Circadian and clock-gene mechanisms	("circadian"[Title] OR "clock gene*"[Title/Abstract] OR "circadian gene*"[Title/Abstract] OR "BMAL1"[Title/Abstract] OR "CLOCK gene"[Title/Abstract] OR "PER1"[Title/Abstract] OR "PER2"[Title/Abstract] OR "CRY1"[Title/Abstract] OR "diurnal"[Title]) AND ("epilep*"[Title/Abstract] OR "seizure*"[Title/Abstract])	260
**Total unique records after duplicate removal**		**3,048**

Field tags: [Title] restricts a term to the article title; [Title/Abstract] searches title and abstract. The asterisk (*) is a truncation wildcard. Counts reflect records retrieved into the reference library at the time of searching; re-running the strings at a later date may return slightly different totals owing to continued database indexing.
